# Patient and kidney transplant survival in type 1 diabetics after kidney transplant alone compared to simultaneous pancreas‐kidney transplant

**DOI:** 10.1111/ans.17663

**Published:** 2022-03-30

**Authors:** James A. Hedley, Patrick J. Kelly, Angela C. Webster

**Affiliations:** ^1^ Collaborative Centre for Organ Donation Evidence, Faculty of Medicine and Health University of Sydney Sydney New South Wales Australia; ^2^ Centre for Transplant and Renal Research Westmead Hospital Sydney New South Wales Australia

**Keywords:** chronic kidney failure, kidney transplantation, pancreas transplantation, survival analysis, type 1 diabetes mellitus

## Abstract

**Background:**

Donor and other differences mean understanding drivers of transplant survival for type 1 diabetics is challenging. We aimed to compare outcomes of simultaneous pancreas‐kidney transplant over kidney transplant alone for people with end‐stage kidney disease (ESKD) and type 1 diabetes.

**Methods:**

We performed a population‐based cohort study comparing outcomes from kidney alone and kidney‐pancreas transplants using registry data. Our study population was people in Australia and New Zealand with type 1 diabetes and ESKD who received a kidney transplant in 1984–2016. Primary outcomes were time to kidney transplant failure and all‐cause death. Secondary outcomes were time to cardiovascular and non‐cardiovascular death. We compared adjusted survival using Cox regression (hazard ratio HR and 95% confidence intervals CI).

**Results:**

Of 1295 type 1 diabetics receiving a transplant, 430 (33%) received deceased donor kidney, 172 (13%) received living donor kidney, and 693 (54%) received pancreas‐kidney transplant. Compared to deceased donor kidney, pancreas‐kidney recipients had 40% lower rate of kidney transplant failure (adjusted HR 0.60; 95% CI 0.45–0.81; *p* = 0.001) and 34% lower mortality (adjusted HR 0.66; 95% CI 0.53–0.83; *p* < 0.001), driven by 49% reduction in cardiovascular mortality (adjusted HR 0.51; 95% CI 0.36–0.72; *p* < 0.001). Pancreas‐kidney recipients had similar reductions in transplant failure and mortality compared to living kidney recipients, after adjusting for transplant timing.

**Conclusions:**

For people with type 1 diabetes, pancreas‐kidney transplant provides improved transplant and overall survival compared to deceased donor kidney alone. Living donor kidneys may perform just as well as pancreas‐kidney transplant if waiting times are short.

## Graphical abstract

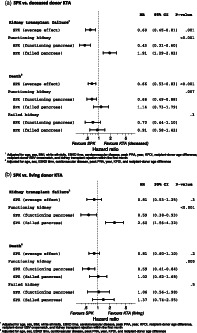

## Introduction

Approximately 1550 people had end‐stage kidney disease (ESKD) and type 1 diabetes mellitus (T1DM) in Australia and New Zealand in 2019.^1^ Kidney transplant is usually the superior treatment option for ESKD, with mortality around 2.3 deaths per 100 person‐years compared to 14 on dialysis.^2^ Transplantation of the whole pancreas (or islet cells) is the only procedure that reverses T1DM.^3^ The first pancreas transplant in Australia and New Zealand was performed in 1984, while the first simultaneous pancreas‐kidney transplant (SPK) was performed in 1987.^4^

Although the benefits of kidney transplant for ESKD and pancreas transplant for T1DM are clear, there is debate about whether SPK is always superior to kidney transplant alone (KTA) in people with ESKD and T1DM. A retrospective cohort study in Italy from 1985 to 2002 found that long‐term kidney transplant survival was greater after SPK compared to KTA in people with T1DM and ESKD.^5^ A prospective cohort study conducted in the USA from 1966 to 1995 found that SPK improved overall survival compared to KTA, potentially due to a reduction in cardiovascular risk.^6^

In the Australia and New Zealand setting, data is available for the whole population from the Australian and New Zealand Dialysis and Transplant Registry (ANZDATA), as well as the Australian and New Zealand Islets and Pancreas Transplant Registry (ANZIPTR). A recent simulation study using data from Australia and New Zealand showed that for patients with T1DM and ESKD, SPK transplantation provided the greatest benefit in terms of patient survival and quality of life compared to kidney transplant alone or dialysis.^7^ However, this analysis did not comprehensively compare SPK with kidney transplant alone. It did not include living kidney donor transplants, did not consider transplant survival as an outcome, and relied on a decision model rather than statistical analysis hence was unable to adjust for important confounding characteristics.

We aimed to determine any benefits of SPK compared to KTA from a deceased donor (D‐KTA) or from a living donor (L‐KTA) in people with ESKD and T1DM in Australia and New Zealand.

## Methods

We performed a population‐based cohort study using data from two transplant registries, ANZDATA and ANZIPTR. Our aim was to compare SPK versus D‐KTA or L‐KTA among people with T1DM and ESKD. Primary outcomes were time to kidney transplant failure and time to all‐cause death, while secondary outcomes were time to cardiovascular and non‐cardiovascular death. Cardiovascular death included any death caused by myocardial ischaemia, haemorrhagic pericarditis, hypertensive cardiac failure, cardiac arrest, cardiac failure, cerebrovascular accident, or aortic aneurysm rupture. Our study population was all people in Australia and New Zealand with T1DM and ESKD who received a kidney transplant between 1st January 1984 and 31st December 2016. This period was chosen because the first pancreas transplant in Australia and New Zealand was in 1984. We excluded people who were under 18 years of age at the time of their first transplant, and people who received other organs along with their kidney/pancreas transplant (e.g., combined kidney, pancreas and liver transplant). We did not explicitly restrict our analysis to people who would be eligible for both a pancreas and/or kidney transplant; rather we adjusted for characteristics with different eligibility criteria for these transplants (e.g., age and BMI). We had ethical oversight from the University of Sydney (project number 2018/515).

We considered characteristics relating to the recipient, the donor, the recipient and donor crossmatch, the transplant, post‐transplant outcomes and drug therapies. The recipient‐level characteristics (measured at time of transplant) were age, sex, body mass index (BMI), time from ESKD onset to transplant (ESKD time), ethnicity, residence remoteness (with New Zealand included as a separate category), country, cause of ESKD, cardiovascular disease, cerebrovascular disease and smoking status. Donor‐level characteristics were age, sex, BMI, cause of death, kidney donor profile index (KDPI),^8^ smoking status, diabetes, hypertension, and cancer. Cross matching characteristics were age difference, sex mismatch, blood group mismatch, peak panel reactive antibodies (PRA), cytomegalovirus (CMV) mismatch and Epstein–Barr virus (EBV) mismatch. Transplant characteristics were transplant era (1984–2000, 2001–2005, 2006–2010 and 2011–2016), and cold ischaemia time. Post‐transplant characteristics were rejection (with or without transplant failure) within the first month, and kidney/pancreas transplant failure. Drug therapies were agents used for antibody induction therapy and agents used for ongoing immunosuppression.

Some characteristics were not collected, or not routinely collected by the registries (i.e., collected sporadically) until after the beginning of the study period: height, weight, cardiovascular disease, cerebrovascular disease and smoking status were first routinely collected in 1993; donor height, weight and cause of death in 1989; recipient CMV and EBV status in 1991; donor CMV status in 1989; and donor EBV status in 1998. Furthermore, donor smoking status, diabetes, and hypertension were first collected in 1993; and donor cancer in 2002. Missing values were determined using multiple imputation.

Remoteness was categorized using the recipient's postcode of residence at ESKD onset and the Accessibility/Remoteness Index of Australia (ARIA) published by the Australian Bureau of Statistics (ABS) and was therefore unavailable for New Zealand recipients. Where postcode was not collected (mostly for records prior to 1993), we instead used the postcode of the referring hospital.

The KDPI is a score from 1% to 100% used to rank deceased donor kidneys in terms of their expected function and risk of failure.^8^ It is based on the KDRI, which is calculated using a formula that accounts for donor age, hypertension, diabetes, creatinine, stroke cause of death, height, weight, and donation after circulatory death (DCD) pathway. Although the KDPI is not intended for use with living donors, for our analysis we calculated KDPI for both living and deceased donors. For the components of the KDRI formula based on cause of death and donation pathway, we conservatively applied the best possible score in these areas to living donors.

Antibody induction therapies were anti T‐cell agents (IV immunoglobulin, muromonab‐CD3, or polyclonal anti T‐cell) or anti interleukin‐2 receptor (anti IL‐2R) agents (basiliximab and daclizumab). Immunosuppression consisted of calcineurin inhibitors (cyclosporin or tacrolimus), anti‐proliferative agents (azathioprine or mycophenolate) or mechanistic target of rapamycin (mTor) inhibitors (sirolimus or everolimus). The combinations of immunosuppression and antibody induction therapy were of particular interest, and the drug combinations we considered were calcineurin inhibitor with anti T‐cell agent, calcineurin inhibitor with anti‐IL‐2R agent, calcineurin inhibitor with no antibody induction therapy, and all other combinations. Since antibody induction therapy was a peri‐transplant intervention, and immunosuppression regimes did not change substantially over time post‐transplant, we only considered the first drug combination used after transplant. In sensitivity analysis we included drug combination as a time‐varying covariate to allow for changes over time.

### Outcomes

The two primary outcomes were time to kidney transplant failure, and time to death. Recipients entered the analysis at the time of their first kidney transplant (KTA or SPK) and were censored at last follow‐up, or if they received a subsequent transplant (kidney and/or pancreas). For kidney transplant survival, we considered both overall transplant survival and death‐censored transplant survival.^9^ Our secondary outcomes were time to cardiovascular death, and time to non‐cardiovascular death. For cardiovascular survival we censored all non‐cardiovascular deaths, and for non‐cardiovascular survival we censored cardiovascular deaths.

### Statistical analysis

Statistical analysis was performed using Stata 15 (StataCorp 2017, College Station, TX). We linked records between ANZDATA and ANZIPTR using transplant date, transplant type, year of birth, sex, blood group, state and date of death. Due to the relatively small number of records in both datasets, we manually verified all linked records.

We compared unadjusted kidney transplant and overall survival between recipients of D‐KTA, L‐KTA and SPK using Kaplan–Meier plots, with *p*‐values calculated using log‐rank tests. For our secondary outcomes we used Nelson‐Aalen cumulative hazard plots to compare unadjusted cause‐specific survival. We performed adjusted analyses using Cox proportional hazards regression models. For each outcome, we considered both the average effect of transplant type (i.e., without time‐varying covariates for kidney/pancreas transplant failure), as well as the time‐varying effect (i.e., including time‐varying covariates for kidney/pancreas transplant failure).

The model building process was performed separately using the average effect models of kidney transplant survival and overall survival. Multivariable models were always adjusted for age, sex, ESKD time, KDPI and recipient‐donor age difference, as these were perceived to have a high probability of being confounders. All other characteristics were added to the initial multivariable model and were only considered for inclusion in the final model if they were confounders (>20% change in any hazard ratio (HR) for transplant type) or were potentially associated with survival (*p* < 0.25). Starting with the highest *p*‐value, we then sequentially removed characteristics from the multivariable model if they were not confounders (<20% change in any hazard ratio (HR) for transplant type) or were not important predictors (*p* > 0.05). Finally, we assessed interactions between each characteristic and transplant type, and kept these in the model if there was evidence of an interaction effect (*p* < 0.01). The proportional hazards assumption was assessed globally, and goodness of fit was assessed using Cox‐Snell residuals.^10^

### Sensitivity analysis

For the secondary outcomes of cardiovascular and non‐cardiovascular death, we performed a sensitivity analysis where we analysed cause‐specific mortality using the Fine and Grey competing risks model.^11^ We were only able to perform this sensitivity analysis on the average effect model, since time‐varying covariates included in the Fine and Grey competing risks model could introduce bias in the effect estimates.^12^

We also performed a sensitivity analysis comparing overall survival from the start of ESKD (initiation on dialysis) instead of time from first transplant, which allowed inclusion of people who never received a transplant. Since most characteristics included in the multivariable models were related to the donor and transplant, only unadjusted survival was considered in the sensitivity analysis.

To understand the impact of imputing missing values in our dataset, we performed a sensitivity analysis where incomplete cases were excluded from the multivariable models.

## Results

**Fig. 1:**
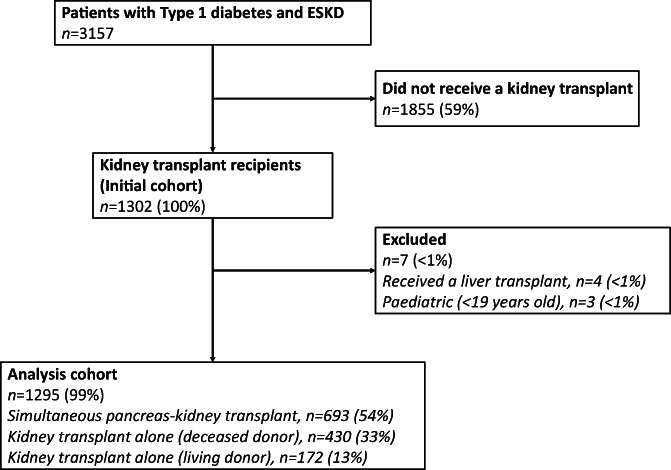
Flowchart of patients included in the analysis; Australia and New Zealand 1984–2016.

The people included in the analysis are shown in Figure 1. Of 1302 people with T1DM and ESKD who received a transplant, we excluded 4 (<1%) who received a combined kidney and liver transplant and 3 (<1%) who were less than 18 years old at the time of their first transplant. Among 1295 kidney transplant recipients in the study cohort, 716 (55%) also received a pancreas transplant (alone or together with a kidney transplant) and all 716 (100%) were linked to ANZIPTR. There were 430 (33%) who initially received D‐KTA, 172 (13%) who initially received L‐KTA and 693 (54%) who initially received SPK.

SPK recipients were more likely to be younger, female, lower BMI and white, compared to KTA recipients (Supplementary Table 1). They also had shorter waiting times between ESKD and first transplant, with 47% receiving a transplant within the first year compared to 31% for D‐KTA recipients.

### Model selection

Results from the univariable analyses are presented in Supplementary Table 4, and results from the bivariable analyses (adjusted for transplant type) are presented in Supplementary Table 5.

The average effect model for kidney transplant survival was adjusted for age, sex, white ethnicity, BMI, cerebrovascular disease, ESKD time, peak PRA, transplant era, KDPI, donor smoking status, recipient‐donor age difference, recipient‐donor EBV crossmatch and kidney transplant rejection within the first month. The average effect model for overall survival was adjusted for age, sex, cardiovascular disease, ESKD time, peak PRA, transplant era, KDPI and recipient‐donor age difference. No additional characteristics were important predictors or confounders. There was no evidence against the proportional hazard's assumptions in the adjusted models for kidney transplant survival or overall survival (*p* > 0.05). Cox‐Snell residual plots indicated the models fit the data well.

### Kidney transplant survival

Of 1295 people who received a kidney transplant, 309 (24%) experienced kidney transplant failure over a total of 9350 person‐years of follow‐up, and median time to kidney failure was 18.1 years (Inter‐quartile range IQR 10.1–25.4). Considering death as a kidney transplant failure there were 604 failures (47%), and median time to kidney failure or death was 11.5 years (IQR 6.2–18.4).

**Fig. 2:**
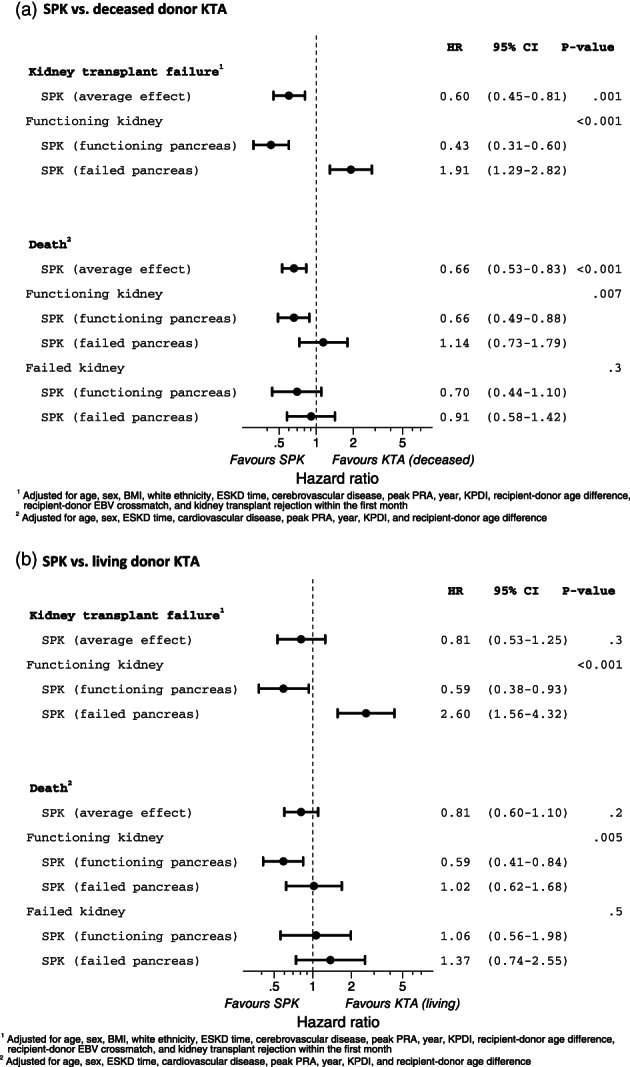
Forest plot of survival after simultaneous pancreas‐kidney transplant compared to kidney transplant alone. (a) SPK versus deceased donor KTA. (b) SPK versus living donor KTA.

Kaplan–Meier plots of kidney transplant survival are presented in Supplementary Figure 1. The unadjusted analyses suggest there is a strong association between transplant type and kidney transplant survival (*p* < 0.001). The results of the adjusted analyses are presented in Figure 2. On average, SPK recipients had a 40% lower rate of kidney transplant failure compared to D‐KTA (adjusted HR 0.60; 95% CI 0.45–0.81; *p* = 0.001), but there was no difference between SPK and L‐KTA (adjusted HR 0.81; 95% CI 0.53–1.25; p = 0.3). Results were similar after adjustment for a failed pancreas transplant. Kidney transplant survival was significantly worse (*p* < 0.001) for SPK recipients with a failed pancreas transplant, who had approximately twice the rate of kidney transplant failure compared to recipients of D‐KTA (adjusted HR 1.91; 95% CI 1.29–2.82) and L‐KTA (adjusted HR 2.60; 95% CI 1.56–4.32), indicating the expected correlation between kidney transplant failure and pancreas transplant failure. Results and conclusions were similar when death was included as a kidney transplant failure (Supplementary Table 2).

### Overall survival

Among the 1855 people who never received a transplant, there were 1324 deaths (71%) over a total of 5428 person‐years of follow‐up since start of dialysis, and median survival was 3 years (IQR 1.5–5.3).

In our analysis cohort there were 482 deaths (37%) over a total of 10 224 person‐years of follow‐up, and median survival was 14.5 years (IQR 7.9–21.3). Of these, 228 (47%) were cardiovascular deaths, while 254 (53%) were non‐cardiovascular deaths.

Overall survival is presented in Supplementary Figure 1, while cause‐specific mortality is summarized as cumulative hazards in Supplementary Figure 2. The unadjusted analyses suggest there is a strong association between transplant type and overall survival (*p* < 0.001), which appears to be driven by better cardiovascular survival among SPK recipients. The results of the adjusted analyses of overall survival are presented in Figure 2. On average, SPK recipients had a 34% lower mortality rate compared to D‐KTA (adjusted HR 0.66; 95% CI 0.53–0.83; *p* < 0.001), but there was no difference compared to L‐KTA (adjusted HR 0.81; 95% CI 0.60–1.10; p = 0.2). Results were similar after adjustment for failed kidney and pancreas transplants. Unlike kidney transplant survival, there was no difference in overall survival between KTA recipients and SPK recipients with a failed pancreas transplant, and there was no difference between KTA and SPK recipients with a failed kidney transplant.

Results of the cause‐specific analyses of overall survival are presented in Supplementary Table 3. The overall survival benefit is primarily driven by a 49% reduction in the rate of cardiovascular mortality for SPK recipients compared to D‐KTA (adjusted HR 0.51; 95% CI 0.36–0.72; *p* < 0.001), while there was no difference in terms of non‐cardiovascular mortality (adjusted HR 0.83; 95% CI 0.61–1.12; *p* = 0.2). Results were similar after adjustment for kidney and pancreas transplant failure.

### Sensitivity analysis

Results and conclusions are similar to the cause‐specific Cox regression models, and when drug combination is included as a time‐varying covariate (Supplementary Table 6).

Unadjusted overall survival from the start of ESKD is presented in the Kaplan–Meier plot in Supplementary Figure 3. Any kidney transplant greatly improves survival (*p* < 0.001). SPK was associated with a 47% reduction in mortality compared to D‐KTA (HR 0.53; 95% CI 0.41–0.68), and a 45% reduction in mortality compared to L‐KTA (HR 0.55; 95% CI 0.40–0.76). These results are similar to unadjusted survival measured from first kidney transplant.

The results from the multivariable models based on the 918 (71%) people with complete cases are presented in Supplementary Table 7 for kidney transplant survival, and Supplementary Table 8 for overall survival. Results were consistent with the main analyses based on 1295 (100%) people including imputed values.

## Discussion

We found that on average, kidney transplant survival and overall survival were improved for SPK recipients compared to D‐KTA but are not different compared to L‐KTA after adjusting for waiting time. Improvements in overall survival were driven by reduced cardiovascular mortality for SPK recipients compared to D‐KTA. Kidney and pancreas transplant failures in SPK recipients are highly correlated, hence an SPK recipient who experiences pancreas transplant failure will have much worse kidney transplant survival compared to a D‐KTA recipient who never received a pancreas transplant. There is no difference in overall survival between KTA recipients and SPK recipients with a failed pancreas transplant.

Our results are consistent with studies from the USA and Europe, as well as a recent simulation using Australian data, which also find that SPK is associated with improved kidney transplant survival and overall survival compared to KTA.^5^, ^6^, ^7^ While these studies only looked at single transplant centres, we have included all people in Australia and New Zealand hence our results are more robust to possible selection bias. It is possible that people selected for SPK were different than those who received KTA in ways we have not adjusted for (e.g., better prognosis) which would potentially bias our results in favour of SPK. Similarly, people who receive a L‐KTA will likely be different to those who are unable to find a living donor, but it is unclear in which direction this could potentially bias our results. For example, people who find a living donor may have better outcomes (e.g., due to a better support network), but those who receive a D‐KTA or SPK may also have better outcomes since they have survived long enough to reach the top of the transplant waiting list (i.e., survivorship bias). We have adjusted for a range of clinically important characteristics to minimize the impact of any potential bias.

Although some people had missing data requiring imputation which may have potentially biased our results, sensitivity analysis demonstrated that results were not different to those obtained by excluding incomplete cases, suggesting our findings are robust.

A further limitation is our application of the KDPI to living donors, despite it only being intended for comparison of deceased donor kidneys. It is likely that living donors would be healthier than deceased donors and more closely matched, and hence have higher quality kidneys than indicated by the KDPI. We found that KDPI was not a confounder for the effect of transplant type and was not strongly associated with either kidney transplant or overall survival, so any measurement errors are unlikely to have influenced our results.

Although we found no difference between SPK and L‐KTA, this was after adjustment for waiting time since ESKD. In practice, people who are able to find a living kidney donor will typically have access to a kidney transplant much earlier than those who must wait for a deceased donor. Therefore, where waiting times are uncertain, and a living donor is possible, it is reasonable to pursue living donation. If a living donor cannot be found before a deceased donor becomes available, or if waiting times are short, then SPK should be preferred. Since most people on the pancreas transplant waiting list have T1DM and ESKD, a pancreas from a deceased donor should be allocated together with a kidney from the same donor where possible. Although the rate of kidney transplant failure is higher in SPK recipients who have experienced pancreas failure, on average the kidney will function for longer when transplanted together with a pancreas instead of alone.

Further research could explore other important differences in outcomes between living donor KTA and SPK, such as waiting time and transplant rejection. This would require more granular data than is typically possible with registry or administrative health data. There is also potential to explore integration of patient preferences and quality of life impacts of the trade‐off between waiting time and post‐transplant outcomes.

## Conclusions

People with T1DM and ESKD have improved kidney transplant survival and overall survival after transplant with SPK compared to D‐KTA. Outcomes are similar for SPK and L‐KTA. Improvements in overall survival in SPK recipients appear to be driven by an improvement in cardiovascular mortality, with no difference in non‐cardiovascular mortality. Kidney transplant failure in SPK recipients is much more likely after experiencing pancreas transplant failure because both organs tend to fail together, however kidneys will function for longer on average when transplanted together with a pancreas instead of alone.

## Author contributions

**James A. Hedley** Formal analysis; investigation; methodology; writing – original draft; writing – review & editing. **Patrick J. Kelly:** Formal analysis; methodology; writing – review and editing. **Angela C. Webster:** Conceptualization; methodology; writing – review and editing.

## Conflict of interest

None declared.

## Data sharing and accessibility

The data used in this study are available on request to the authors, with permission from the Australian and New Zealand Dialysis and Transplant Registry, and the Australian and New Zealand Islet and Pancreas Transplant Registry.

## Funding

The authors declare no external funding was received for this work.

## Supporting information


**Supplementary Figure 1** Kaplan–Meier plots of unadjusted kidney transplant survival and overall survival.
Supplementary Figure 1(a): Kidney transplant survival.
Supplementary Figure 1(b): Overall survival.

**Supplementary Figure 2** Nelson‐Aalen cumulative hazard plots of cause‐specific mortality.
Supplementary Figure 2(a): Cardiovascular mortality.
Supplementary Figure 2(b): Non‐cardiovascular mortality.

**Supplementary Figure 3** Sensitivity analysis—Kaplan–Meier plot of unadjusted overall survival from start of end‐stage kidney disease.

**Appendix** S1: Supporting Information.
