# Supplementary figures and images for: Patient and kidney transplant survival in type 1 diabetics after kidney transplant alone compared to simultaneous pancreas‐kidney transplant
Source: ANZ J Surg. 2022 Mar 30;92(7-8):1856–62. doi: 10.1111/ans.17663 (PMC9543845; doi:10.1111/ans.17663)

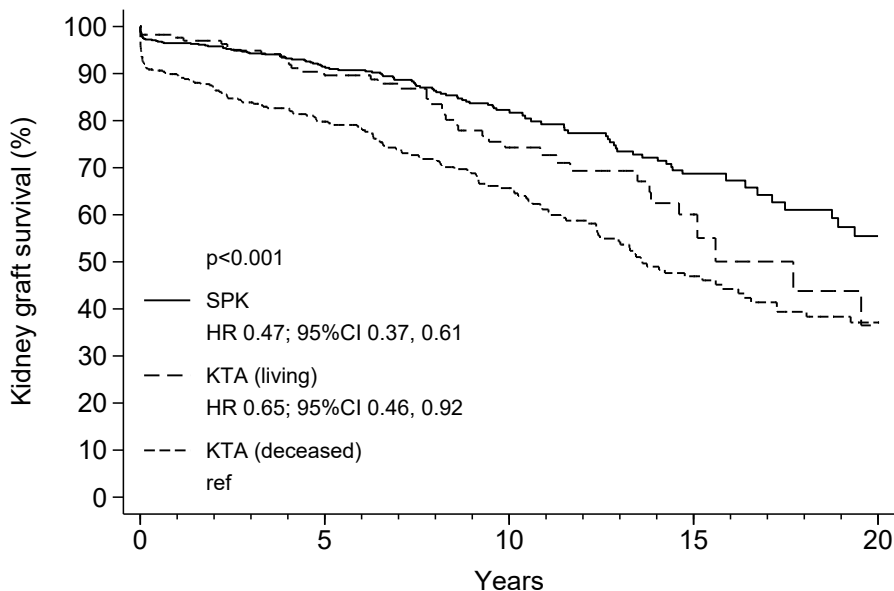

| No. of patients |     |     |     |    |    |
|-----------------|-----|-----|-----|----|----|
| KTA (deceased)  | 430 | 235 | 133 | 65 | 29 |
| KTA (living)    | 172 | 113 | 54  | 21 | 5  |
| SPK             | 693 | 389 | 191 | 82 | 28 |

Supplement: Supplementary file 1 — Supplementary Figure 1 Kaplan–Meier plots of unadjusted kidney transplant survival and overall survival. Supplementary Figure 1(a): Kidney transplant survival. Supplementary Figure 1(b): Overall survival. [file ANS-92-1856-s001.zip › ANS_17663_Supplementary Figure 1A.pdf]

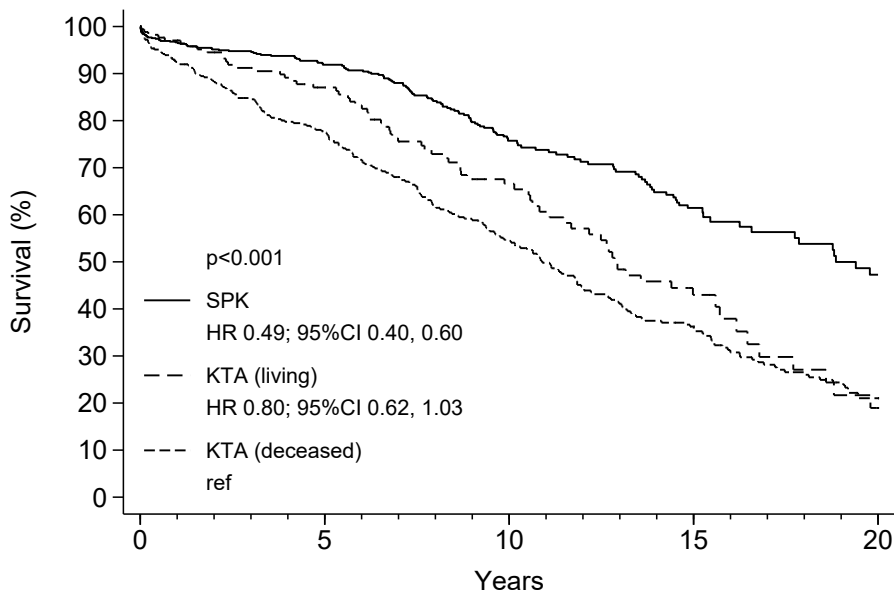

| No. of patients |     |     |     |    |    |
|-----------------|-----|-----|-----|----|----|
| KTA (deceased)  | 430 | 259 | 155 | 83 | 37 |
| KTA (living)    | 172 | 119 | 61  | 27 | 6  |
| SPK             | 693 | 411 | 211 | 94 | 32 |

Supplement: Supplementary file 1 — Supplementary Figure 1 Kaplan–Meier plots of unadjusted kidney transplant survival and overall survival. Supplementary Figure 1(a): Kidney transplant survival. Supplementary Figure 1(b): Overall survival. [file ANS-92-1856-s001.zip › ANS_17663_Supplementary Figure 1B.pdf]

Cardiovascular death (%)

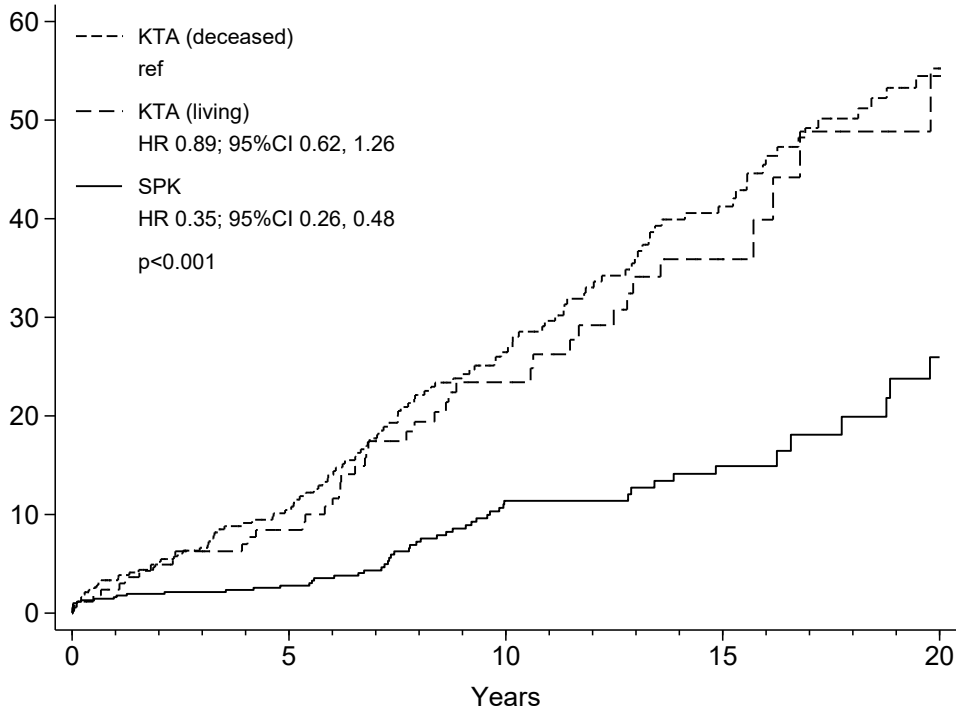

Supplement: Supplementary file 2 — Supplementary Figure 2 Nelson‐Aalen cumulative hazard plots of cause‐specific mortality. Supplementary Figure 2(a): Cardiovascular mortality. Supplementary Figure 2(b): Non‐cardiovascular mortality. [file ANS-92-1856-s003.zip › ANS_17663_Supplementary Figure 2A.pdf]

Non-cardiovascular death (%)

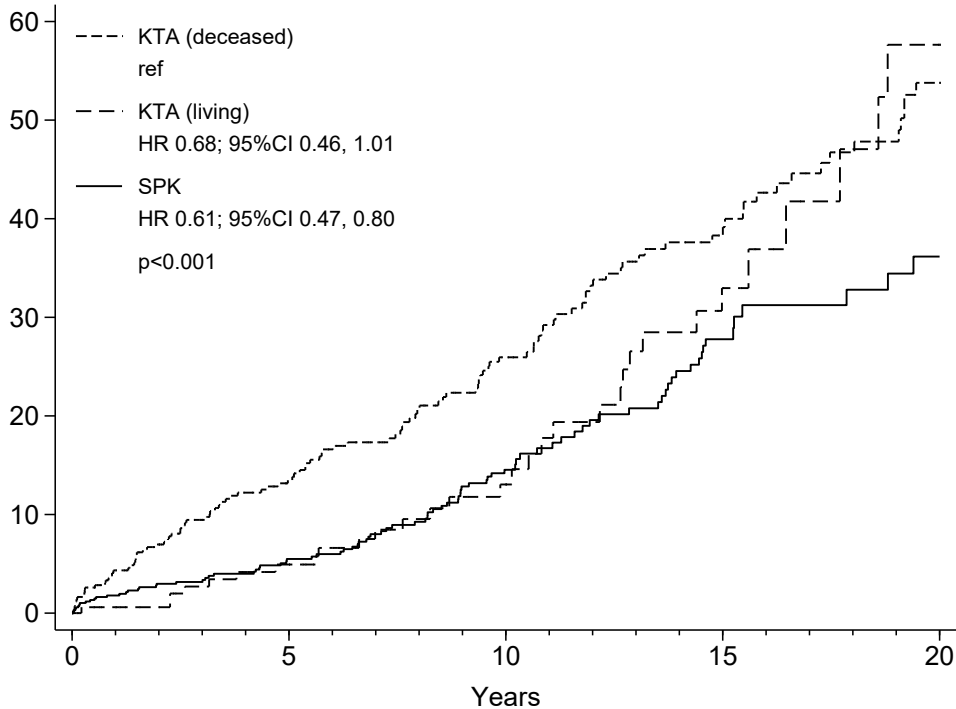

Supplement: Supplementary file 2 — Supplementary Figure 2 Nelson‐Aalen cumulative hazard plots of cause‐specific mortality. Supplementary Figure 2(a): Cardiovascular mortality. Supplementary Figure 2(b): Non‐cardiovascular mortality. [file ANS-92-1856-s003.zip › ANS_17663_Supplementary Figure 2B.pdf]

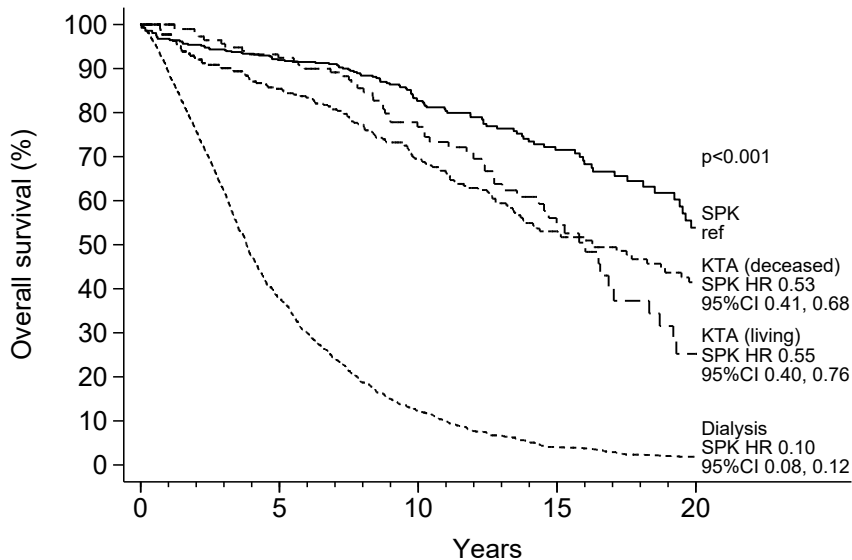

| No. of patients |       |     |     |     |    |
|-----------------|-------|-----|-----|-----|----|
| Dialysis        | 2,962 | 416 | 101 | 49  | 23 |
| KTA (deceased)  | 12    | 257 | 186 | 80  | 37 |
| KTA (living)    | 38    | 117 | 72  | 33  | 8  |
| SPK             | 137   | 436 | 251 | 104 | 33 |

Supplement: Supplementary file 3 — Supplementary Figure 3 Sensitivity analysis—Kaplan–Meier plot of unadjusted overall survival from start of end‐stage kidney disease. [file ANS-92-1856-s004.pdf]
