# Supplementary material for: Patient and kidney transplant survival in type 1 diabetics after kidney transplant alone compared to simultaneous pancreas‐kidney transplant
Source: ANZ J Surg. 2022 Mar 30;92(7-8):1856–62. doi: 10.1111/ans.17663 (PMC9543845; doi:10.1111/ans.17663)
Supplement: Supplementary file 4 — Appendix S1: Supporting Information. [file ANS-92-1856-s002.docx]

# Supplementary figures

Supplementary Figure 1: Kaplan-Meier plots of unadjusted kidney transplant survival and overall survival

Supplementary Figure 1A: Kidney transplant survival

Supplementary Figure 1B: Overall survival

Supplementary Figure 2: Nelson-Aalen cumulative hazard plots of cause-specific mortality

Supplementary Figure 2A: Cardiovascular mortality

Supplementary Figure 2B: Non-cardiovascular mortality

Supplementary Figure 3: Sensitivity analysis – Kaplan-Meier plot of unadjusted overall survival from start of end-stage kidney disease

# Supplementary tables

Supplementary Table 1: Baseline characteristics at time of first kidney transplant

|  | **First kidney transplant, n (column %)** | | | | | | | | |
| --- | --- | --- | --- | --- | --- | --- | --- | --- | --- |
|  | KTA (Deceased) | | KTA (Living) | | SPK | | Total | | P-value |
| **Total** | 430 | (33) | 172 | (13) | 693 | (54) | 1,295 | (100) |  |
| **Age, mean (SD)** | 43.9 | (10.1) | 43.3 | (10.6) | 38.6 | (7.3) | 41 | (9.2) | <0.001 |
| 18-24 | 10 | (2) | 3 | (2) | 5 | (<1) | 18 | (1) |  |
| 25-34 | 75 | (17) | 36 | (21) | 227 | (33) | 338 | (26) |  |
| 35-44 | 137 | (32) | 61 | (35) | 302 | (44) | 500 | (39) |  |
| 45-54 | 130 | (30) | 40 | (23) | 152 | (22) | 322 | (25) |  |
| 55+ | 78 | (18) | 32 | (19) | 7 | (1) | 117 | (9) |  |
| **Sex** |  |  |  |  |  |  |  |  | 0.003 |
| Female | 155 | (36) | 72 | (42) | 322 | (46) | 549 | (42) |  |
| Male | 275 | (64) | 100 | (58) | 371 | (54) | 746 | (58) |  |
| **BMI (kg/m2), mean (SD)** | 25.6 | (4.7) | 25.4 | (4.5) | 24.5 | (3.8) | 24.9 | (4.2) | <0.001 |
| Underweight (0-18.4) | 6 | (2) | 4 | (2) | 17 | (3) | 27 | (2) |  |
| Normal or Overweight (18.5-29.9) | 251 | (84) | 138 | (84) | 590 | (90) | 979 | (88) |  |
| Obese (30+) | 41 | (14) | 23 | (14) | 48 | (7) | 112 | (10) |  |
| *Not collected* | 132 | - | 7 | - | 38 | - | 177 | - |  |
| **Years from ESKD onset to transplant, median (IQR)** | 1.7 | (0.8, 2.9) | 0.8 | (0.2, 1.8) | 1.1 | (0.3, 2.2) | 1.3 | (0.4, 2.4) | <0.001 |
| <1 year | 135 | (31) | 92 | (53) | 328 | (47) | 555 | (43) |  |
| 1-2 years | 117 | (27) | 40 | (23) | 174 | (25) | 331 | (26) |  |
| 2-3 years | 78 | (18) | 13 | (8) | 103 | (15) | 194 | (15) |  |
| 3+ years | 100 | (23) | 27 | (16) | 88 | (13) | 215 | (17) |  |
| **Ethnicity** |  |  |  |  |  |  |  |  | 0.001 |
| Indigenous Australian | 8 | (2) | 1 | (<1) | 6 | (<1) | 15 | (1) |  |
| Maori/Pacific Islander | 25 | (6) | 5 | (3) | 12 | (2) | 42 | (3) |  |
| White | 379 | (88) | 159 | (92) | 661 | (95) | 1,199 | (93) |  |
| Asian | 11 | (3) | 4 | (2) | 3 | (<1) | 18 | (1) |  |
| Indian | 3 | (<1) | 3 | (2) | 6 | (<1) | 12 | (<1) |  |
| African and Middle Eastern | 0 | (0) | 0 | (0) | 4 | (<1) | 4 | (<1) |  |
| Other | 1 | (<1) | 0 | (0) | 1 | (<1) | 2 | (<1) |  |
| **Country** |  |  |  |  |  |  |  |  | <0.001 |
| Australia | 351 | (82) | 143 | (83) | 643 | (93) | 1,137 | (88) |  |
| New Zealand | 79 | (18) | 29 | (17) | 50 | (7) | 158 | (12) |  |
| **Remoteness (Australia only)** |  |  |  |  |  |  |  |  | 0.1 |
| Major city | 254 | (72) | 100 | (70) | 455 | (71) | 809 | (71) |  |
| Inner regional | 60 | (17) | 23 | (16) | 125 | (19) | 208 | (18) |  |
| Outer regional | 36 | (10) | 15 | (10) | 58 | (9) | 109 | (10) |  |
| Remote | 1 | (<1) | 4 | (3) | 4 | (<1) | 9 | (<1) |  |
| Very remote | 0 | (0) | 1 | (<1) | 1 | (<1) | 2 | (<1) |  |
| **Peak PRA %, median (IQR)** | 0 | (0, 10) | 0 | (0, 3) | 0 | (0, 6) | 0 | (0, 7) | 0.01 |
| <50 | 395 | (92) | 164 | (95) | 663 | (96) | 1,222 | (94) |  |
| 50+ | 35 | (8) | 8 | (5) | 30 | (4) | 73 | (6) |  |
| **Transplant year** |  |  |  |  |  |  |  |  | <0.001 |
| 1984-2000 | 255 | (59) | 58 | (34) | 169 | (24) | 482 | (37) |  |
| 2001-2005 | 59 | (14) | 42 | (24) | 133 | (19) | 234 | (18) |  |
| 2006-2010 | 41 | (10) | 37 | (22) | 165 | (24) | 243 | (19) |  |
| 2011-2016 | 75 | (17) | 35 | (20) | 226 | (33) | 336 | (26) |  |
| **Cause of ESKD** |  |  |  |  |  |  |  |  | <0.001 |
| Type 1 diabetes | 406 | (94) | 155 | (90) | 676 | (98) | 1,237 | (96) |  |
| Glomerulonephritis | 16 | (4) | 10 | (6) | 9 | (1) | 35 | (3) |  |
| Other^†^ | 8 | (2) | 7 | (4) | 8 | (1) | 23 | (2) |  |
| **Cardiovascular disease** |  |  |  |  |  |  |  |  | <0.001 |
| No | 210 | (59) | 93 | (55) | 483 | (71) | 786 | (65) |  |
| Yes | 145 | (41) | 76 | (45) | 197 | (29) | 418 | (35) |  |
| *Not collected* | 75 | - | 3 | - | 13 | - | 91 | - |  |
| **Cerebrovascular disease** |  |  |  |  |  |  |  |  | <0.001 |
| No | 321 | (92) | 154 | (169) | 652 | (107) | 1,127 | (93) |  |
| Yes | 28 | (8) | 15 | (16) | 26 | (4) | 69 | (6) |  |
| *Not collected* | 81 | - | 3 | - | 15 | - | 99 | - |  |
| **Smoking status** |  |  |  |  |  |  |  |  | <0.001 |
| Never | 152 | (48) | 86 | (52) | 397 | (59) | 635 | (55) |  |
| Former | 112 | (35) | 59 | (36) | 205 | (31) | 376 | (33) |  |
| Current | 56 | (18) | 21 | (13) | 68 | (10) | 145 | (13) |  |
| *Not collected* | 110 | - | 6 | - | 23 | - | 139 | - |  |
| **Donor's Age, mean (SD)** | 37.9 | (18.1) | 45.9 | (16.3) | 26.9 | (9.8) | 33.1 | (15.7) | <0.001 |
| 0-44 years | 252 | (59) | 65 | (38) | 666 | (96) | 983 | (76) |  |
| 45+ years | 177 | (41) | 106 | (62) | 27 | (4) | 310 | (24) |  |
| *Not collected* | 1 | - | 1 | - | 0 | - | 2 | - |  |
| **Donor's Sex** |  |  |  |  |  |  |  |  | <0.001 |
| Female | 189 | (44) | 93 | (58) | 259 | (37) | 541 | (42) |  |
| Male | 237 | (56) | 67 | (42) | 433 | (63) | 737 | (58) |  |
| *Not collected* | 4 | - | 12 | - | 1 | - | 17 | - |  |
| **Donor's BMI (kg/m2), mean (SD)** | 25.6 | (5.5) | 27.9 | (7.9) | 24.1 | (3.7) | 24.8 | (4.9) | <0.001 |
| Underweight (0-18.4) | 18 | (6) | 0 | (0) | 32 | (5) | 50 | (5) |  |
| Normal or Overweight (18.5-29.9) | 250 | (79) | 65 | (76) | 618 | (91) | 933 | (86) |  |
| Obese (30+) | 47 | (15) | 20 | (24) | 32 | (5) | 99 | (9) |  |
| *Height or weight not collected* | 115 | - | 87 | - | 11 | - | 213 | - |  |
| **Donor's cause of death** |  |  |  |  |  |  |  |  | <0.001 |
| Intracranial haemorrhage | 160 | (46) | - |  | 200 | (29) | 360 | (30) |  |
| Traumatic brain injury | 97 | (28) | - |  | 334 | (48) | 431 | (36) |  |
| Cerebral infarct | 14 | (4) | - |  | 26 | (4) | 40 | (3) |  |
| Cerebral hypoxia/ischaemia | 53 | (15) | - |  | 98 | (14) | 151 | (12) |  |
| Other neurological condition | 3 | (<1) | - |  | 3 | (<1) | 6 | (<1) |  |
| Non-neurological condition | 21 | (6) | - |  | 32 | (5) | 53 | (4) |  |
| *Not collected* | 82 | - |  |  | 0 | - | 82 | - |  |
| **Donor's relationship** |  |  |  |  |  |  |  |  | <0.001 |
| Deceased | 430 | (100) | 0 | (0) | 693 | (100) | 1,123 | (87) |  |
| Living related | 0 | (0) | 105 | (61) | 0 | (0) | 105 | (8) |  |
| Living unrelated | 0 | (0) | 67 | (39) | 0 | (0) | 67 | (5) |  |
| **KDPI**^‡^**, median (IQR)** | 31 | (13, 54) | 34 | (23.5, 55.5) | 13 | (4, 25) | 21 | (8, 36) | <0.001 |
| 0-24 | 180 | (42) | 49 | (28) | 517 | (75) | 746 | (58) |  |
| 25-49 | 127 | (30) | 73 | (42) | 168 | (24) | 368 | (28) |  |
| 50-74 | 74 | (17) | 43 | (25) | 8 | (1) | 125 | (10) |  |
| 75-100 | 49 | (11) | 7 | (4) | 0 | (0) | 56 | (4) |  |
| **Donor's smoking status** |  |  |  |  |  |  |  |  | <0.001 |
| Never | 150 | (55) | 122 | (74) | 313 | (48) | 585 | (54) |  |
| Former | 31 | (11) | 35 | (21) | 47 | (7) | 113 | (10) |  |
| Current | 92 | (34) | 7 | (4) | 293 | (45) | 392 | (36) |  |
| *Not collected* | 157 | - | 8 | - | 40 | - | 205 | - |  |
| **Donor diabetes** |  |  |  |  |  |  |  |  | <0.001 |
| No | 262 | (96) | 161 | (98) | 693 | (100) | 1,116 | (99) |  |
| Yes | 11 | (4) | 3 | (2) | 0 | (0) | 14 | (1) |  |
| *Not collected* | 157 | - | 8 | - | 0 | - | 165 | - |  |
| **Donor hypertension** |  |  |  |  |  |  |  |  | <0.001 |
| No | 238 | (87) | 157 | (96) | 639 | (98) | 1,034 | (95) |  |
| Yes | 35 | (13) | 7 | (4) | 14 | (2) | 56 | (5) |  |
| *Not collected* | 157 | - | 8 | - | 40 | - | 205 | - |  |
| **Donor cancer** |  |  |  |  |  |  |  |  | <0.001 |
| No | 154 | (97) | 105 | (100) | 495 | (99) | 754 | (99) |  |
| Yes | 5 | (3) | 0 | (0) | 5 | (1) | 10 | (1) |  |
| *Not collected* | 271 | - | 67 | - | 193 | - | 531 | - |  |
| **Recipient-donor age difference, mean (SD)** | 6.0 | (19.2) | -2.7 | (16.8) | 11.7 | (12.2) | 7.9 | (16.2) | <0.001 |
| Donor 5+ years younger | 228 | (53) | 37 | (22) | 499 | (72) | 764 | (59) |  |
| Within 5 years | 66 | (15) | 57 | (33) | 119 | (17) | 242 | (19) |  |
| Donor 5-14 years older | 65 | (15) | 36 | (21) | 64 | (9) | 165 | (13) |  |
| Donor 15+ years older | 70 | (16) | 41 | (24) | 11 | (2) | 122 | (9) |  |
| *Donor age not collected* | 1 | - | 1 | - | 0 | - | 2 | - |  |
| **Recipient-donor sex crossmatch** |  |  |  |  |  |  |  |  | <0.001 |
| Same sex | 210 | (49) | 66 | (41) | 387 | (56) | 663 | (52) |  |
| Different sex | 216 | (51) | 94 | (59) | 305 | (44) | 615 | (48) |  |
| *Donor sex not collected* | 4 | - | 12 | - | 1 | - | 17 | - |  |
| **Recipient-donor blood group crossmatch** |  |  |  |  |  |  |  |  | <0.001 |
| Same blood group | 387 | (92) | 128 | (78) | 653 | (95) | 1,168 | (92) |  |
| Different blood group | 33 | (8) | 37 | (22) | 37 | (5) | 107 | (8) |  |
| *Recipient or donor blood group not collected* | 10 | - | 7 | - | 3 | - | 20 | - |  |
| **Recipient-donor CMV crossmatch** |  |  |  |  |  |  |  |  | <0.001 |
| Neither recipient nor donor positive | 44 | (14) | 68 | (41) | 125 | (19) | 237 | (21) |  |
| Both recipient and donor positive | 111 | (36) | 0 | (0) | 236 | (36) | 347 | (31) |  |
| Only donor positive | 75 | (24) | 0 | (0) | 156 | (24) | 231 | (21) |  |
| Only recipient positive | 77 | (25) | 98 | (59) | 135 | (21) | 310 | (28) |  |
| *Recipient or donor CMV status not collected* | 123 | - | 6 | - | 41 | - | 170 | - |  |
| **Recipient-donor EBV crossmatch** |  |  |  |  |  |  |  |  | <0.001 |
| Recipient and donor negative | 21 | (10) | 46 | (28) | 71 | (13) | 138 | (15) |  |
| Recipient and donor positive | 106 | (51) | 0 | (0) | 292 | (52) | 398 | (43) |  |
| Recipient negative, donor positive | 15 | (7) | 0 | (0) | 51 | (9) | 66 | (7) |  |
| Recipient positive, donor negative | 65 | (31) | 120 | (72) | 147 | (26) | 332 | (36) |  |
| *Recipient or donor EBV status not collected* | 223 | - | 6 | - | 132 | - | 361 | - |  |
| **Kidney cold ischaemia time, median (IQR)** | 14 | (10, 19) | 2 | (1, 3.5) | 10 | (7, 13) | 10 | (6, 14) | <0.001 |
| 0-5 hours | 61 | (14) | 159 | (92) | 87 | (13) | 307 | (24) |  |
| 6-11 hours | 78 | (18) | 13 | (8) | 363 | (52) | 454 | (35) |  |
| 12-17 hours | 154 | (36) | 0 | (0) | 217 | (31) | 371 | (29) |  |
| 18+ hours | 137 | (32) | 0 | (0) | 26 | (4) | 163 | (13) |  |
| **Immunosuppression and antibody induction therapy** |  |  |  |  |  |  |  |  | <0.001 |
| Calcineurin inhibitor + Anti T-cell | 48 | (11) | 13 | (8) | 98 | (14) | 159 | (12) |  |
| Calcineurin inhibitor + Anti IL-2R | 111 | (26) | 76 | (44) | 338 | (49) | 525 | (41) |  |
| Calcineurin inhibitor only | 25 | (6) | 3 | (2) | 1 | (<1) | 29 | (2) |  |
| Other combination | 246 | (57) | 80 | (47) | 256 | (37) | 582 | (45) |  |

KTA, kidney transplant alone; SPK, simultaneous pancreas-kidney transplant; ESKD, end-stage kidney disease; SD, standard deviation; IQR, interquartile range; BMI, body mass index; PRA, panel reactive antibodies; CMV, cytomegalovirus; EBV, Epstein-Barr virus, Anti IL-2R, anti interleukin-2 receptor

^†^ Other causes of ESKD include: congenital renal hypoplasia and dysplasia, cortical necrosis, haemolytic uraemic syndrome, interstitial nephritis, nephrosclerosis, oxalosis, polycystic kidney disease, posterior urethral valves, and reflux nephropathy

^‡^ KDPI is intended for assessing the quality of kidneys from deceased donors, however we have applied the same formula to living donors as well. Living donor kidneys are likely better quality than suggested by the KDPI

Supplementary Table 2: Multivariable Cox proportional hazards regression models – Kidney transplant survival

|  | **Censored at death** | | |  | **Including death** | | |
| --- | --- | --- | --- | --- | --- | --- | --- |
| **Model** | *HR* | *95% CI* | *p-value* |  | *HR* | *95% CI* | *p-value* |
| **SPK vs. KTA (deceased donor)** |  |  |  |  |  |  |  |
| Average effect | 0.60 | 0.45, 0.81 | 0.001 |  | 0.67 | 0.54, 0.82 | <0.001 |
| Adjusted for transplant failure |  |  | <0.001 |  |  |  | <0.001 |
| *Functioning pancreas* | 0.43 | 0.31, 0.60 |  |  | 0.55 | 0.44, 0.68 |  |
| *Failed pancreas* | 1.91 | 1.29, 2.82 |  |  | 1.43 | 1.06, 1.93 |  |
| **SPK vs. KTA (living donor)** |  |  |  |  |  |  |  |
| Average effect | 0.81 | 0.53, 1.25 | 0.3 |  | 0.76 | 0.57, 1.02 | 0.06 |
| Adjusted for transplant failure |  |  | <0.001 |  |  |  | <0.001 |
| *Functioning pancreas* | 0.59 | 0.38, 0.93 |  |  | 0.63 | 0.46, 0.85 |  |
| *Failed pancreas* | 2.60 | 1.56, 4.32 |  |  | 1.63 | 1.14, 2.35 |  |

Adjusted for age, sex, BMI, white ethnicity, ESKD time, cerebrovascular disease, peak PRA, year, KDPI, recipient-donor age difference, recipient-donor EBV crossmatch, and rejection within first month

Supplementary Table 3: Multivariable Cox proportional hazards regression models for overall survival and cause-specific survival

|  | **All-cause death** | | |  | **Cardiovascular death** | | |  | **Non-cardiovascular death** | | |
| --- | --- | --- | --- | --- | --- | --- | --- | --- | --- | --- | --- |
| **Model** | *HR* | *95% CI* | *p-value* |  | *HR* | *95% CI* | *p-value* |  | *HR* | *95% CI* | *p-value* |
| **SPK vs. KTA (deceased donor)** |  |  |  |  |  |  |  |  |  |  |  |
| Average effect | 0.66 | 0.53, 0.83 | <0.001 |  | 0.51 | 0.36, 0.72 | <0.001 |  | 0.83 | 0.61, 1.12 | 0.2 |
| Adjusted for transplant failure |  |  |  |  |  |  |  |  |  |  |  |
| Functioning kidney |  |  | 0.007 |  |  |  | 0.004 |  |  |  | 0.5 |
| *Functioning pancreas* | 0.66 | 0.49, 0.88 |  |  | 0.51 | 0.32, 0.80 |  |  | 0.81 | 0.55, 1.18 |  |
| *Failed pancreas* | 1.14 | 0.73, 1.79 |  |  | 1.28 | 0.68, 2.40 |  |  | 1.03 | 0.54, 1.97 |  |
| Failed kidney |  |  | 0.3 |  |  |  | 0.03 |  |  |  | 0.9 |
| *Functioning pancreas* | 0.70 | 0.44, 1.10 |  |  | 0.32 | 0.13, 0.76 |  |  | 1.13 | 0.64, 1.98 |  |
| *Failed pancreas* | 0.91 | 0.58, 1.42 |  |  | 0.78 | 0.40, 1.51 |  |  | 1.05 | 0.57, 1.94 |  |
| **SPK vs. KTA (living donor)** |  |  |  |  |  |  |  |  |  |  |  |
| Average effect | 0.81 | 0.60, 1.10 | 0.2 |  | 0.54 | 0.35, 0.83 | 0.005 |  | 1.20 | 0.77, 1.86 | 0.4 |
| Adjusted for transplant failure |  |  |  |  |  |  |  |  |  |  |  |
| Functioning kidney |  |  | 0.005 |  |  |  | <0.001 |  |  |  | 0.7 |
| *Functioning pancreas* | 0.59 | 0.41, 0.84 |  |  | 0.37 | 0.22, 0.62 |  |  | 0.90 | 0.54, 1.50 |  |
| *Failed pancreas* | 1.02 | 0.62, 1.68 |  |  | 0.93 | 0.47, 1.85 |  |  | 1.15 | 0.55, 2.40 |  |
| Failed kidney |  |  | 0.5 |  |  |  | 0.2 |  |  |  | 0.3 |
| *Functioning pancreas* | 1.06 | 0.56, 1.98 |  |  | 0.44 | 0.16, 1.25 |  |  | 1.92 | 0.80, 4.57 |  |
| *Failed pancreas* | 1.37 | 0.74, 2.55 |  |  | 1.07 | 0.45, 2.56 |  |  | 1.79 | 0.73, 4.37 |  |

Adjusted for age, sex, ESKD time, cardiovascular disease, peak PRA, year, KDPI, and recipient-donor age difference

Supplementary Table 4: Univariable Cox-proportional hazards regression models

|  | **Kidney transplant survival** | | | **Overall survival** | | |
| --- | --- | --- | --- | --- | --- | --- |
|  | Censored at death | | | All-cause mortality | | |
| **Characteristic** | *HR* | *95% CI* | *p-value* | *HR* | *95% CI* | *p-value* |
| **Transplant type** |  |  | <0.001 |  |  | <0.001 |
| KTA (Deceased) | ref | - |  | ref | - |  |
| KTA (Living) | 0.65 | 0.46, 0.92 |  | 0.80 | 0.62, 1.03 |  |
| SPK | 0.47 | 0.37, 0.61 |  | 0.49 | 0.40, 0.60 |  |
| **Age (per 5 years)** | 0.97 | 0.91, 1.04 | 0.4 | 1.27 | 1.20, 1.34 | <0.001 |
| **Sex** |  |  | 0.02 |  |  | 0.1 |
| Female | ref | - |  | ref | - |  |
| Male | 0.77 | 0.62, 0.97 |  | 1.15 | 0.96, 1.37 |  |
| **BMI (per 5 kg/m^2^)** | 1.17 | 1.03, 1,32 | 0.02 | 1.11 | 1.00, 1.23 | 0.05 |
| **ESKD time (per year)** | 1.02 | 0.95, 1.09 | 0.6 | 1.16 | 1.10, 1.21 | <0.001 |
| **White** | 0.59 | 0.40, 0.86 | 0.006 | 0.77 | 0.55, 1.07 | 0.1 |
| **Country** |  |  | 0.2 |  |  | 0.4 |
| Australia | ref | - |  | ref | - |  |
| New Zealand | 1.26 | 0.94, 1.70 |  | 1.11 | 0.87, 1.41 |  |
| **Remoteness (within Australia)** |  |  | 0.7 |  |  | 0.3 |
| Major city | ref | - |  | ref | - |  |
| Regional | 0.89 | 0.67, 1.18 |  | 0.83 | 0.66, 1.04 |  |
| Remote | 0.89 | 0.28, 2.78 |  | 0.87 | 0.36, 2.10 |  |
| **Peak PRA (%)** |  |  | 0.04 |  |  | 0.006 |
| <50 | ref | - |  | ref | - |  |
| 50+ | 1.52 | 1.03, 2.26 |  | 1.54 | 1.13, 2.10 |  |
| **Transplant year** |  |  | <0.001 |  |  | 0.04 |
| 1984-2000 | ref | - |  | ref | - |  |
| 2001-2005 | 0.74 | 0.55, 1.00 |  | 0.72 | 0.56, 0.93 |  |
| 2006-2010 | 0.45 | 0.29, 0.70 |  | 0.77 | 0.56, 1.05 |  |
| 2011-2016 | 0.50 | 0.30, 0.84 |  | 0.72 | 0.45, 1.15 |  |
| **Cause of ESKD** |  |  | 0.6 |  |  | 0.4 |
| Type 1 diabetes | ref | - |  | ref | - |  |
| Glomerulonephritis | 1.37 | 0.73, 2.57 |  | 1.00 | 0.56, 1.77 |  |
| Other | 0.98 | 0.41, 2.38 |  | 0.49 | 0.18, 1.31 |  |
| **Cardiovascular disease** | 1.36 | 1.08, 1.72 | 0.009 | 1.81 | 1.51, 2.17 | <0.001 |
| **Cerebrovascular disease** | 1.55 | 0.98, 2.48 | 0.06 | 2.06 | 1.49, 2.85 | <0.001 |
| **Smoking status** |  |  | 0.3 |  |  | 0.2 |
| Never | ref | - |  | ref | - |  |
| Former | 0.82 | 0.63, 1.06 |  | 1.05 | 0.86, 1.29 |  |
| Current | 1.04 | 0.75, 1.44 |  | 1.26 | 0.98, 1.62 |  |
| **Donor's Age (per 5 years)** | 1.12 | 1.08, 1.16 | <0.001 | 1.09 | 1.06, 1.12 | <0.001 |
| **Donor's Sex** |  |  | 0.3 |  |  | 0.2 |
| Female | ref | - |  | ref | - |  |
| Male | 0.89 | 0.71, 1.11 |  | 0.89 | 0.75, 1.07 |  |
| **Donor's BMI (per 5 kg/m2)** | 1.16 | 1.04, 1.30 | 0.009 | 1.19 | 1.08, 1.31 | <0.001 |
| **Donor's cause of death** |  |  | <0.001 |  |  | 0.08 |
| Living donor | ref | - |  | ref | - |  |
| Intracranial haemorrhage | 1.46 | 1.03, 2.08 |  | 1.08 | 0.82, 1.41 |  |
| Traumatic brain injury | 0.87 | 0.60, 1.25 |  | 0.81 | 0.61, 1.06 |  |
| Cerebral infarct | 1.06 | 0.53, 2.11 |  | 0.86 | 0.48, 1.52 |  |
| Cerebral hypoxia/ischaemia | 0.52 | 0.27, 0.98 |  | 0.65 | 0.41, 1.03 |  |
| Other neurological condition | - |  |  | 1.22 | 0.17, 8.85 |  |
| Non-neurological condition | 0.94 | 0.50, 1.75 |  | 0.82 | 0.50, 1.35 |  |
| **KDPI (per 5 percentage points)** | 1.07 | 1.04, 1.09 | <0.001 | 1.05 | 1.03, 1.07 | <0.001 |
| **Donor's smoking status** |  |  | 0.08 |  |  | 0.1 |
| Never | ref | - |  | ref | - |  |
| Former | 0.98 | 0.61, 1.58 |  | 1.41 | 1.02, 1.94 |  |
| Current | 1.30 | 1.03, 1.64 |  | 1.02 | 0.83, 1.24 |  |
| **Donor diabetes** | 0.90 | 0.29, 2.81 | 0.9 | 2.28 | 1.22, 4.28 | 0.01 |
| **Donor hypertension** | 1.64 | 1.05, 2.55 | 0.03 | 1.07 | 0.71, 1.63 | 0.7 |
| **Donor cancer** | 2.27 | 1.01, 5.10 | 0.05 | 1.28 | 0.53, 3.10 | 0.6 |
| **Recipient-donor age difference (per 5 years)** | 0.90 | 0.87, 0.93 | <0.001 | 0.99 | 0.96, 1.01 | 0.3 |
| **Recipient-donor sex crossmatch** |  |  | 0.1 |  |  | 0.02 |
| Same sex | ref | - |  | ref | - |  |
| Different sex | 1.20 | 0.96, 1.50 |  | 1.23 | 1.03, 1.47 |  |
| **Recipient-donor blood group crossmatch** |  |  | 0.6 |  |  | 0.6 |
| Same blood group | ref | - |  | ref | - |  |
| Different blood group | 0.91 | 0.62, 1.34 |  | 1.08 | 0.81, 1.44 |  |
| **Recipient-donor CMV crossmatch** |  |  | 0.3 |  |  | 0.4 |
| Neither recipient nor donor positive | ref | - |  | ref | - |  |
| Both recipient and donor positive | 0.92 | 0.68, 1.26 |  | 1.21 | 0.94, 1.56 |  |
| Only donor positive | 1.24 | 0.88, 1.73 |  | 1.16 | 0.87, 1.55 |  |
| Only recipient positive | 1.12 | 0.82, 1.54 |  | 1.25 | 0.96, 1.63 |  |
| **Recipient-donor EBV crossmatch** |  |  | 0.02 |  |  | 0.05 |
| Neither recipient nor donor positive | ref | - |  | ref | - |  |
| Both recipient and donor positive | 0.75 | 0.54, 1.04 |  | 0.72 | 0.54, 0.95 |  |
| Only donor positive | 0.55 | 0.29, 1.04 |  | 0.67 | 0.40, 1.13 |  |
| Only recipient positive | 0.69 | 0.54, 0.90 |  | 0.82 | 0.67, 1.00 |  |
| **Kidney cold ischaemia time (per 5 hours)** | 1.02 | 0.94, 1.11 | 0.7 | 1.05 | 0.99, 1.12 | 0.1 |
| **Rejection within first month** | 1.15 | 0.84, 1.57 | 0.4 | 0.84 | 0.65, 1.10 | 0.2 |
| **Immunosuppression and antibody induction therapy** | | | <0.001 |  |  | <0.001 |
| Calcineurin inhibitor + Anti T-cell | ref | - |  | ref | - |  |
| Calcineurin inhibitor + Anti IL-2R | 0.69 | 0.46, 1.02 |  | 0.85 | 0.58, 1.23 |  |
| Calcineurin inhibitor only | 1.81 | 1.13, 2.91 |  | 1.01 | 0.59, 1.71 |  |
| Other combination | 1.16 | 0.85, 1.57 |  | 1.72 | 1.31, 2.27 |  |

Supplementary Table 5: Bivariable Cox-proportional hazards regression models for kidney transplant survival and overall survival

|  | **Kidney transplant survival** | | | **Overall survival** | | |
| --- | --- | --- | --- | --- | --- | --- |
|  | Censored at death | | | All-cause mortality | | |
| **Characteristic** | *HR* | *95% CI* | *p-value* | *HR* | *95% CI* | *p-value* |
| **Age (per 5 years)** | 0.94 | 0.88, 1.00 | 0.05 | 1.23 | 1.16, 1.29 | <0.001 |
| **Female** | 1.37 | 1.09, 1.71 | 0.06 | 0.93 | 0.77, 1.12 | 0.4 |
| **BMI (per 5 kg/m^2^)** | 1.16 | 1.02, 1.31 | 0.02 | 1.09 | 0.99, 1.21 | 0.08 |
| **ESKD time (per year)** | 0.99 | 0.92, 1.06 | 0.7 | 1.13 | 1.08, 1.18 | <0.001 |
| **White** | 0.69 | 0.47, 1.01 | 0.06 | 0.91 | 0.66, 1.27 | 0.6 |
| **Country** |  |  | 0.7 |  |  | 0.6 |
| Australia | ref | - |  | ref | - |  |
| New Zealand | 1.07 | 0.79, 1.45 |  | 0.93 | 0.73, 1.20 |  |
| **Remoteness** |  |  | 0.9 |  |  | 0.4 |
| Major city | ref | - |  | ref | - |  |
| Regional | 0.91 | 0.68, 1.20 |  | 0.82 | 0.65, 1.03 |  |
| Remote | 1.05 | 0.33, 3.31 |  | 0.92 | 0.38, 2.25 |  |
| New Zealand | 1.04 | 0.76, 1.42 |  | 0.89 | 0.69, 1.14 |  |
| **Peak PRA (%)** |  |  | 0.1 |  |  | 0.04 |
| <50 | ref | - |  | ref | - |  |
| 50+ | 1.35 | 0.90, 2.00 |  | 1.40 | 1.02, 1.91 |  |
| **Transplant year** |  |  | 0.04 |  |  | 0.6 |
| 1984-2000 | ref | - |  | ref | - |  |
| 2001-2005 | 0.85 | 0.63, 1.15 |  | 0.84 | 0.65, 1.09 |  |
| 2006-2010 | 0.57 | 0.37, 0.88 |  | 0.97 | 0.70, 1.35 |  |
| 2011-2016 | 0.61 | 0.36, 1.02 |  | 0.89 | 0.55, 1.43 |  |
| **Cause of ESKD** |  |  | 0.8 |  |  | 0.2 |
| Type 1 diabetes | ref | - |  | ref | - |  |
| Glomerulonephritis | 1.23 | 0.65, 2.32 |  | 0.88 | 0.48, 1.51 |  |
| Other | 0.90 | 0.37, 2.18 |  | 0.43 | 0.16, 1.16 |  |
| **Cardiovascular disease** | 1.33 | 1.05, 1.68 | 0.02 | 1.76 | 1.46, 2.12 | <0.001 |
| **Cerebrovascular disease** | 1.49 | 0.93, 2.37 | 0.1 | 2.04 | 1.47, 2.83 | <0.001 |
| **Smoking status** |  |  | 0.1 |  |  | 0.7 |
| Never | ref | - |  | ref | - |  |
| Former | 0.76 | 0.58, 0.99 |  | 0.98 | 0.79, 1.20 |  |
| Current | 0.89 | 0.64, 1.23 |  | 1.10 | 0.85, 1.41 |  |
| **Donor's Age (per 5 years)** | 1.10 | 1.06, 1.14 | <0.001 | 1.06 | 1.03, 1.09 | <0.001 |
| **Donor female** | 1.04 | 0.83, 1.31 | 0.7 | 1.04 | 0.87, 1.25 | 0.7 |
| **Donor's BMI (per 5 kg/m2)** | 1.13 | 1.01, 1.26 | 0.04 | 1.15 | 1.05, 1.26 | 0.003 |
| **Donor's cause of death** |  |  | 0.002 |  |  | 0.3 |
| Living donor | ref | - |  | ref | - |  |
| Intracranial haemorrhage | 1.54 | 0.87, 2.72 |  | 1.26 | 0.79, 2.00 |  |
| Traumatic brain injury | 1.03 | 0.58, 1.84 |  | 1.08 | 0.68, 1.72 |  |
| Cerebral infarct | 1.25 | 0.55, 2.86 |  | 1.13 | 0.57, 2.24 |  |
| Cerebral hypoxia/ischaemia | 0.56 | 0.26, 1.24 |  | 0.78 | 0.43, 1.42 |  |
| Other neurological condition | - |  |  | 1.48 | 0.20, 11.10 |  |
| Non-neurological condition | - |  |  | - |  |  |
| **KDPI (per 5 percentage points)** | 1.05 | 1.02, 1.08 | <0.001 | 1.02 | 1.00, 1.05 | 0.02 |
| **Donor's smoking status** |  |  | 0.02 |  |  | 0.1 |
| Never | ref | - |  | ref | - |  |
| Former | 0.98 | 0.61, 1.58 |  | 1.40 | 1.01, 1.93 |  |
| Current | 1.41 | 1.11, 1.80 |  | 1.10 | 0.90, 1.35 |  |
| **Donor diabetes** | 0.66 | 0.21, 2.06 | 0.5 | 1.73 | 0.92, 3.26 | 0.09 |
| **Donor hypertension** | 1.33 | 0.85, 2.08 | 0.2 | 0.89 | 0.58, 1.35 | 0.6 |
| **Donor cancer** | 1.88 | 0.84, 4.24 | 0.1 | 1.12 | 0.46, 2.70 | 0.8 |
| **Recipient-donor age difference (per 5 years)** | 0.91 | 0.88, 0.94 | <0.001 | 1.01 | 0.98, 1.03 | 0.7 |
| **Recipient-donor sex crossmatch** |  |  | 0.3 |  |  | 0.1 |
| Same sex | ref | - |  | ref | - |  |
| Different sex | 1.13 | 0.90, 1.42 |  | 1.16 | 0.97, 1.39 |  |
| **Recipient-donor blood group crossmatch** |  |  | 0.4 |  |  | 0.9 |
| Same blood group | ref | - |  | ref | - |  |
| Different blood group | 0.86 | 0.58, 1.27 |  | 1.02 | 0.76, 1.36 |  |
| **Recipient-donor CMV crossmatch** |  |  | 0.3 |  |  | 0.3 |
| Neither recipient nor donor positive | ref | - |  | ref | - |  |
| Both recipient and donor positive | 0.90 | 0.65, 1.25 |  | 1.28 | 0.98, 1.68 |  |
| Only donor positive | 1.19 | 0.84, 1.70 |  | 1.21 | 0.89, 1.64 |  |
| Only recipient positive | 1.09 | 0.79, 1.50 |  | 1.22 | 0.94, 1.59 |  |
| **Recipient-donor EBV crossmatch** |  |  | 0.1 |  |  | 0.4 |
| Neither recipient nor donor positive | ref | - |  | ref | - |  |
| Both recipient and donor positive | 0.90 | 0.65, 1.26 |  | 0.88 | 0.66, 1.18 |  |
| Only donor positive | 0.66 | 0.34, 1.26 |  | 0.80 | 0.48, 1.34 |  |
| Only recipient positive | 0.73 | 0.57, 0.95 |  | 0.86 | 0.70, 1.05 |  |
| **Kidney cold ischaemia time (per 5 hours)** | 0.94 | 0.86, 1.03 | 0.2 | 1.01 | 0.94, 1.08 | 0.8 |
| **Rejection within first month** | 1.35 | 0.98, 1.87 | 0.07 | 1.02 | 0.78, 1.34 | 0.9 |
| **Immunosuppression and antibody induction therapy** | | | 0.05 |  |  | <0.001 |
| Calcineurin inhibitor + Anti T-cell | ref | - |  | ref | - | ref |
| Calcineurin inhibitor + Anti IL-2R | 0.67 | 0.45, 0.99 |  | 0.80 | 0.55, 1.16 | 0.67 |
| Calcineurin inhibitor only | 1.32 | 0.81, 2.16 |  | 0.73 | 0.43, 1.26 | 1.32 |
| Other combination | 0.96 | 0.70, 1.32 |  | 1.39 | 1.05, 1.85 | 0.96 |

Supplementary Table 6: Sensitivity analysis - Fine and Gray competing risks model for cause-specific mortality

|  | **Cardiovascular death** | | |  | **Non-cardiovascular death** | | |
| --- | --- | --- | --- | --- | --- | --- | --- |
| **Model** | *SHR* | *95% CI* | *p-value* |  | *SHR* | *95% CI* | *p-value* |
| SPK vs. KTA (deceased donor) | 0.54 | 0.38, 0.77 | <0.001 |  | 0.90 | 0.66, 1.22 | 0.5 |
| SPK vs. KTA (living donor) | 0.51 | 0.34, 0.78 | 0.002 |  | 1.35 | 0.89, 2.05 | 0.2 |

Adjusted for age, sex, ESKD time, cardiovascular disease, peak PRA, year, KDPI, and recipient-donor age difference

Supplementary Table 7: Sensitivity analysis – Multivariable Cox proportional hazards regression model of kidney transplant survival excluding incomplete cases

|  | **Censored at death** | | |  | **Including death** | | |
| --- | --- | --- | --- | --- | --- | --- | --- |
| **Model** | *HR* | *95% CI* | *p-value* |  | *HR* | *95% CI* | *p-value* |
| **SPK vs. KTA (deceased donor)** |  |  |  |  |  |  |  |
| Average effect | 0.77 | 0.47, 1.25 | 0.3 |  | 0.76 | 0.54, 1.06 | 0.1 |
| Adjusted for transplant failure |  |  | 0.04 |  |  |  | <0.001 |
| *Functioning pancreas* | 0.57 | 0.34, 0.96 |  |  | 0.62 | 0.44, 0.89 |  |
| *Failed pancreas* | 2.24 | 1.23, 4.05 |  |  | 1.63 | 1.05, 2.52 |  |
| **SPK vs. KTA (living donor)** |  |  |  |  |  |  |  |
| Average effect | 0.80 | 0.46, 1.36 | 0.4 |  | 0.76 | 0.53, 1.10 | 0.2 |
| Adjusted for transplant failure |  |  | <0.001 |  |  |  | <0.001 |
| *Functioning pancreas* | 0.60 | 0.34, 1.06 |  |  | 0.64 | 0.43, 0.93 |  |
| *Failed pancreas* | 2.34 | 1.23, 4.45 |  |  | 1.66 | 1.04, 2.65 |  |

Adjusted for age, sex, BMI, white ethnicity, ESKD time, cerebrovascular disease, peak PRA, year, KDPI, recipient-donor age difference, recipient-donor EBV crossmatch, and rejection within first month

Supplementary Table 8: Sensitivity analysis – Multivariable Cox proportional hazards regression model of overall survival excluding incomplete cases

|  | **All-cause death** | | |  | **Cardiovascular death** | | |  | **Non-cardiovascular death** | | |
| --- | --- | --- | --- | --- | --- | --- | --- | --- | --- | --- | --- |
| **Model** | *HR* | *95% CI* | *p-value* |  | *HR* | *95% CI* | *p-value* |  | *HR* | *95% CI* | *p-value* |
| **SPK vs. KTA (deceased donor)** |  |  |  |  |  |  |  |  |  |  |  |
| Average effect | 0.96 | 0.65, 1.42 | 0.8 |  | 0.83 | 0.44, 1.57 | 0.6 |  | 1.06 | 0.65, 1.74 | 0.8 |
| Adjusted for transplant failure |  |  |  |  |  |  |  |  |  |  |  |
| Functioning kidney |  |  | 0.2 |  |  |  | 0.03 |  |  |  | 0.9 |
| *Functioning pancreas* | 0.84 | 0.53, 1.34 |  |  | 0.64 | 0.30, 1.37 |  |  | 1.01 | 0.56, 1.81 |  |
| *Failed pancreas* | 1.52 | 0.78, 2.93 |  |  | 2.16 | 0.84, 5.55 |  |  | 1.12 | 0.44, 2.85 |  |
| Failed kidney |  |  | 0.5 |  |  |  | 0.5 |  |  |  | 0.4 |
| *Functioning pancreas* | 1.41 | 0.68, 2.94 |  |  | 0.52 | 0.10, 2.79 |  |  | 1.83 | 0.78, 4.29 |  |
| *Failed pancreas* | 1.42 | 0.68, 2.96 |  |  | 1.48 | 0.45, 4.90 |  |  | 1.41 | 0.55, 3.57 |  |
| **SPK vs. KTA (living donor)** |  |  |  |  |  |  |  |  |  |  |  |
| Average effect | 0.85 | 0.58, 1.25 | 0.4 |  | 0.50 | 0.28, 0.89 | 0.02 |  | 1.34 | 0.79, 2.29 | 0.3 |
| Adjusted for transplant failure |  |  |  |  |  |  |  |  |  |  |  |
| Functioning kidney |  |  | 0.05 |  |  |  | 0.003 |  |  |  | 0.9 |
| *Functioning pancreas* | 0.63 | 0.40, 0.99 |  |  | 0.34 | 0.17, 0.68 |  |  | 1.04 | 0.56, 1.95 |  |
| *Failed pancreas* | 1.13 | 0.59, 2.17 |  |  | 1.15 | 0.47, 2.80 |  |  | 1.16 | 0.44, 3.03 |  |
| Failed kidney |  |  | 0.9 |  |  |  | 0.3 |  |  |  | 0.4 |
| *Functioning pancreas* | 1.08 | 0.50, 2.33 |  |  | 0.25 | 0.05, 1.31 |  |  | 2.01 | 0.76, 5.31 |  |
| *Failed pancreas* | 1.09 | 0.50, 2.34 |  |  | 0.71 | 0.22, 2.29 |  |  | 1.54 | 0.54, 4.38 |  |

Adjusted for age, sex, ESKD time, cardiovascular disease, peak PRA, year, KDPI, and recipient-donor age difference
